# Supplementary material for: Arm weight effects on dynamic walking stability in individuals with hemiparetic stroke
Source: PLoS One. 2024 Dec 17;19(12):e0314463. doi: 10.1371/journal.pone.0314463 (PMC11651544; doi:10.1371/journal.pone.0314463)
Supplement: Attachment — Submitted filename: Response to Reviewers.docx [file pone.0314463.s002.docx]

Authors’ Response to Reviewers (In Blue)

Reviewers' comments:

Reviewer #1: This study examined the effects of arm weights on dynamic stability during overground walkingin individuals with hemiparetic stroke. The results indicated that arm weights could be a viable addition to rehabilitation programs, potentially enhancing proprioceptive feedback and supporting motor recovery without compromising stability. More explanation of the analysis method is needed before publication.

1) Why did the authors use the FSR-based balance evaluation method among the many dynamic stabiilty measures? For example, MOS could be used (or is simpler), but it is necessary to explain the reasons why this method (FSR) was used, including the advantages of FSR for this study.

Both the Feasible Stability Region (FSR) and Margin of Stability (MOS) methods are widely used to assess dynamic stability. MOS is straightforward and commonly used to predict balance loss at key gait events by determining if the COM will exceed the BOS. In contrast, FSR accounts for both forward and backward balance loss and offers greater sensitivity in predicting stability during complex tasks, capturing multi-directional aspects of movement beyond simple linear projections (Yang & Pai, 2014).

Compared with other metrics quantifying dynamic stability, such as Floquet multipliers, Lyapunov exponents, and MOS, FSR-based stability is more closely related to fall risk, as it focuses on maintaining an appropriate COM-BOS relationship. This makes FSR particularly suitable for populations with neurological impairments, such as stroke survivors, where subtle changes in balance control are critical for preventing falls.

(Revised manuscript text) In the “Instrumentation and data analysis” section, we will include the rationale for using FSR: “The FSR method was chosen because, compared to other stability measures like Floquet multipliers, Lyapunov exponents, and margin of stability, FSR is more sensitive to fall risk as it directly evaluates the critical relationship between the COM and BOS, making it especially useful for assessing dynamic stability in complex tasks [24].”

2) Please provide any rationale for the mass of the weight (0.45 kg).

The rationale for using 0.45 kg weights was based on previous research indicating that lighter arm weights can improve walking performance in both healthy individuals and those affected by stroke. Studies have shown that applying lighter weights can enhance walking speed, step length, and cadence by improving proprioceptive feedback and contributing to passive mechanical energy transfer. Specifically, the choice of 0.45 kg weights was informed by prior work showing these enhancements without overwhelming participants or causing significant discomfort. Therefore, we selected this weight to evaluate its effects on dynamic stability in a controlled yet practical manner.

(Revised manuscript text) In the “Methods” section, we will include the rationale for using 0.45 kg weights: “A 0.45 kg sandbag weight was chosen based on studies indicating that lighter weights improve walking performance through enhanced proprioceptive feedback and passive mechanical energy transfer [16, 17]. The weight was selected to provide a manageable perturbation while ensuring participant comfort.”

3) The reason why the light weight did not have much effect on the balance this time may be due to the position of the weight. Participants carried a weight by his/her hand, but the position of the weight is close to whole body COM, so it may not have much effect on dynamic balance. If the position and length of the weight were different, the dynamic balance would be more affected. This point should be included in the discussion.

We agree that the proximity of the weights to the COM could have influenced the results, potentially limiting their effect on dynamic balance. However, since participants were swinging their arms during walking, the weights were not consistently positioned near the COM. Instead, the arm movement caused the weights to shift throughout the gait cycle. This dynamic motion likely distributed the influence of the weights across different phases of gait, reducing their overall effect on stability. This point will be addressed in the discussion.

(Revised manuscript text) In the “Discussion” section, we will add the following statement: “It is also possible that the proximity of the weights to the COM contributed to the limited effect on dynamic balance. However, because participants were swinging their arms during walking, the weights were not fixed in one location near the COM. Instead, the movement of the arms caused the position of the weights to vary throughout the gait cycle, likely dispersing their influence on stability. This dynamic motion may have lessened the overall impact of the weights on balance. Future studies could explore conditions where arm movement is restricted or weights are placed in different locations to better assess their influence on dynamic stability.”

Reviewer #2: I have few concerns connected with this paper:

- Patients: the group is very heterogeneous (the authors correctly listed it as one of the limitations). Two factors of this heterogeneity could influence the results of this study: type and duration of the rehabilitation after stroke received of the patients, and the fact that some of them are over 60 years of age – the natural process of aging starts around this age. This can be the reason of the variability of the effect of weight addition among subjects.

We acknowledge that the group was heterogeneous in terms of rehabilitation history and age, and these factors could indeed contribute to variability in responses to the arm weights. Participants with different types and durations of rehabilitation may have developed unique compensatory strategies, and those over 60 years of age could experience age-related declines in balance and proprioception. However, since we employed a repeated measures design, each participant served as their own control, which reduces the impact of individual differences on the overall findings. Despite this, the heterogeneity may still have contributed to variability within individual responses across conditions, which warrants further exploration in future studies.

(Revised manuscript text) In the “Discussion (Limitations)” section, we will add: “We also acknowledge that the heterogeneity of the participant group, including differences in rehabilitation history and age, could have contributed to the variability in responses. Participants with varied rehabilitation experiences may have developed different compensatory strategies, and those over 60 years of age could experience age-related declines in balance and proprioception. However, because we used a repeated measures design, where each participant served as their own control, the individual differences were minimized in their effect on overall outcomes. Still, this variability may have influenced individual responses across conditions.”

- 3D gait analysis is prone to errors due to the marker placement errors and soft tissues artefacts. From the described methodology I presume that some of the markers were placed on the shirts and shorts adding the errors connected with their movement relative to the patients’ body. This fact can be an additional source of the variability of the obtained results.

We agree that soft tissue artifact and marker placement errors are common challenges in 3D gait analysis. To minimize these issues, we followed consistent marker placement protocols and ensured that markers were placed directly on the skin rather than on clothing. While some error is unavoidable in any motion capture system, these factors likely contributed to variability in the results. However, they are unlikely to have introduced systematic bias across conditions. Future studies may benefit from advanced motion capture techniques, such as markerless systems, to further reduce these sources of error.

(Revised manuscript text) In the “Discussion (Limitations)” section, we will add: “Additionally, potential errors due to soft tissue artifacts and marker placement may have contributed to variability in the results. Although markers were placed directly on the skin following established protocols, such errors are common in 3D gait analysis. Advanced techniques, such as markerless motion capture, should be considered to minimize these issues in future studies.”

- In the Discussion section the authors presented a contradictive (to me) conclusions: that there was no effects of the addition of the weights, and that the analysis of the effect in the individual patients showed in some of them their influence. In my opinion these conclusions should be reformulated.

We understand that the conclusions may initially seem contradictory. However, while no significant changes in stability were found at the group level, individual participants responded differently to the weighted conditions. Some improved, while others experienced decreased or unchanged stability. This reflects the variability in rehabilitation outcomes for stroke survivors and underscores the need for personalized approaches rather than generalized interventions. To avoid any confusion, we will revise our discussion to clarify this point and highlight the importance of considering individual variability in future research and clinical practice.

(Revised manuscript text) In the “Discussion (Conclusion)” section, we will revise the concluding statements: “In conclusion, our findings indicate that the use of 0.45 kg arm weights did not significantly affect the dynamic stability of individuals with hemiparetic stroke at the group level during over-ground walking. However, individual analyses revealed variability in responses, with some participants maintaining or improving stability and others experiencing decreases. This underscores the importance of personalized rehabilitation strategies, as group-level analyses may mask meaningful individual differences. These findings suggest that arm weights could be a viable addition to rehabilitation programs, potentially enhancing proprioceptive feedback and supporting motor recovery without compromising stability. This stability maintenance, coupled with improved walking performance in some individuals, highlights the potential utility of arm weights as an intervention strategy [16].”

- I wonder if some analysis could be performed to reveal which factors influence the patients’ response to the weights.

We appreciate the suggestion to explore factors that might influence individual responses to the arm weights. Factors such as age, rehabilitation history, and baseline motor function may explain the variability observed in this study. Although the small sample size limited our ability to perform detailed analyses, future studies with larger cohorts should investigate whether these factors predict responsiveness to arm weight interventions.

(Revised manuscript text) In the “Discussion” section, we will add: “Furthermore, while our study was limited by its small sample size, further research should investigate whether factors such as age, rehabilitation history, and baseline motor function predict individual responses to arm weight interventions. Identifying these predictors could enable clinicians to tailor rehabilitation programs and enhance the effectiveness of interventions for stroke survivors.”
